# Involvement of CB2 signalling pathway in the development of osteoporosis by regulating the proliferation and differentiation of hBMSCs

**DOI:** 10.1111/jcmm.16128

**Published:** 2021-01-29

**Authors:** Feng Tian, Hong‐tao Yang, Tao Huang, Feng‐feng Chen, Fu‐jun Xiong

**Affiliations:** ^1^ Department of Orthopaedics Xi'an International Medical Center Hospital Xi'an China; ^2^ Department of Orthopaedics The First Affiliated Hospital of Xi'an Medical University Xi'an China

**Keywords:** CB2, human bone marrow stem cells, Notch, osteoporosis

## Abstract

The aim of the present study was to explore the potential mechanism underlying the involvement of CB2 in osteoporosis. Micro‐CT was utilized to examine femur bone architecture. Also, real‐time PCR and Western blot analysis were utilized to detect the effect of 2‐AG on the expression of CB2 and Notch, or the interaction between CB2 and Notch 2. 2‐AG treatment up‐regulated BMD, Tb.Sp and SMI in OVX mice, whereas proportion of bone volume in total volume (BV/TV), trabecular thickness (Tb.Th), trabecular number (Tb.N) and bone mineral density (BMD) were decreased in 2‐AG‐treated OVX mice. Accordingly, 2‐AG administration up‐regulated Notch 1 expression in OVX mice but had no effect on CB2 and Notch 2 expression. Meanwhile, 2‐AG administration promoted the differentiation of hBMSCs in OVX mice, while exhibiting no effect on the proliferation of hBMSCs. Furthermore, in the cellular models, 2‐AG treatment also up‐regulated Notch 1 expression but had no effect on CB2 and Notch 2 expression, while Notch 1 shRNA had no effect on CB2 and Notch 2 expression. 2‐AG promoted cell proliferation and differentiation, which were inhibited by Notch 1 shRNA. NICD had no effect on CB2 level but increased Notch 1 expression, and CB2 shRNA decreased CB2 and Notch 1 expression. Finally, CB2 shRNA inhibited cell proliferation and differentiation, whereas NICD promoted proliferation and differentiation of hBMSCs. Our results provided further evidence for the association of CB2 gene with BMD and osteoporosis, and identified CB2 as a promising target for the treatment of osteoporosis.

## 1 INTRODUCTION

According to the description of World Health Organization (WHO), osteoporosis is a progressive disorder featured by microarchitectural deterioration of bone tissues and low bone mass, with a subsequent elevation in bone fragility and proneness to fracture.^1^ Several recent studies explored the differentiation of hBMSCs towards osteoblasts or the differentiation of haematopoietic stem cells towards osteoclasts. Some reports indicated that, compared with cells from non‐osteoporotic controls, hBMSCs from osteoporotic patients have less osteogenic capacity and show adipogenesis instead of osteogenesis.^2^ However, other reports revealed that in both groups, hBMSCs upon chemically triggered differentiation exhibit the same level of differentiation.^3^

The Notch signalling pathway (NSP) is highly conserved and related to apoptosis, self‐renewal potential, and cell‐fate determination.^4^ NSP was identified to be implicated in the proliferation and differentiation of hBMSCs. The study conducted by Ugarte et al indicated that NS suppressed adipocyte differentiation and promoted osteoblast differentiation of hBMSCs.^5^ Moreover, NS was shown to be associated with the differentiation of BMSCs into other types of cells. The levels of a range of neuron‐specific indicators were elevated when the production of Notch 1 was blocked by mNotch‐1 shRNA, indicating that NS plays a critical role in the BMSC differentiation into neurons in vitro.^6^ Other studies also found that NSP induced the differentiation of BMSCs to endothelial cells.^7^ This system is consisted of the relevant degradative enzymes monoacylglycerol lipase and fatty acid amide hydrolase (FAAH), 2‐arachidonoylglycerol (2‐AG), anandamide (AEA), their endogenous ligands, and the G‐protein coupled cannabinoid (CB) receptors CB1 and CB2.^8^ A recent report has shown that endocannabinoids have an effect opposite to that of NS on cholangiocarcinoma growth.^9^ The increased Notch 1 signalling enhances the expression of Hes genes, which may be a mechanism underlying the role of cannabinoids in regulating and maintaining the neural progenitor cell pool.^10^

Persistent bone loss primarily contributes to substantial decrease in bone formation and osteoblast number in majority of osteoporosis patients. It was shown that CB2 triggers aggravated age‐related osteoporosis due to increased bone turnover.^11^ It has been shown that bone loss in ageing mice with CB2 deficiency is related to increased bone resorption coupled to a substantial decrease in one formation and number of osteoblasts.^12^ Consistent with this, in ovariectomized mice, stimulation of peripheral CB2 receptors prevents bone loss by augmenting bone formation indicators.^11^ These results—together with proof demonstrating solid correlation of CB2 polymorphisms with osteoporotic women—indicated that CB2 agonists are promising activators of bone formation in osteoporosis.^13^

It has been reported that CB2 is a regulator of Notch signalling pathway.^9^ The Notch signal has been known to be a regulator of proliferation and differentiation of hBMSCs, and dysregulation of proliferation and differentiation of hBMSCs is believed to play an important role in the development of osteoporosis.^14^, ^15^ At the same time, Notch signalling has been shown to regulate the expression of CB2, so we hypothesized that CB2 and Notch could form a negative feedback loop to maintain the homoeostasis of various components in the system and regulate the proliferation and differentiation of hBMSCs.^16^ In this study, we verified the hypothesis in an animal model of osteoporosis.

## 2 MATERIALS AND METHODS

### 2.1 Establishment of animal model

All animal tests were carried out according to the Principles of Laboratory Animal Care. All animals were 10 weeks old with a body mass of 20 ~ 30 g. For the first in vivo experiment, 24 mice were subdivided into four groups, and a control group of mice (N = 5) received surgery without an ovariectomy. Distilled water supplemented with gum acacia was utilized to treat the mice once a day on the 8th day after the surgery. A total of 19 mice received bilateral oophorectomy, and 4 mice died during the surgery. Seven days post‐ovariectomy (OVX) treatment, 15 mice were divided randomly and evenly into 3 groups of OVX, OVX + DHEA, and OVX + E2 with 5 mice in each group. In the OVX group, saline was utilized to administer the mice intragastrically. In the OVX + DHEA group, DHEA was utilized to administer the mice intragastrically at a final concentration of 5 mg/kg per day. In the OVX + E2 group, oestrogen (17‐β‐estradiol) was utilized to administer the mice intragastrically once a day. After 12 weeks of treatment, the mice were weighed. Then, all mice were sacrificed to collect tissue and blood samples for further analysis. Another in vivo experiment was carried out to examine whether DHEA served as a metabolite or an oestrogen derivative. A total of 40 mice received bilateral oophorectomy, whereas saline was utilized to treat 20 mice and DHEA was utilized to treat the other 20 mice. Saline‐treated OVX mice or DHEA‐treated OVX mice were divided into 4 groups and received the following treatment: The carrier solvent was utilized to treat one group, and 0.04, 0.2 or 2 μg/d of an aromatase inhibitor letrozole was utilized to treat the other 3 groups for 3 months.

### 2.2 Analysis of bone mineral density

For analysis of BMD (bone mineral density) performed following previous protocol,^17^ the left lumbar and femur vertebrae were collected post‐dissection. An animal PIXImus densitometer (Lunar; GE Copr.) was utilized to carry out dual energy X‐ray absorptiometry (DEXA). Uniformity was maintained via selecting a consistent region of interest.

### 2.3 Bone microarchitecture assessment by micro‐CT

Explore Locus SP Pre‐Clinical Specimen Micro‐CT (GE Healthcare Bio‐Sciences) was utilized to scan bone microarchitecture in the distal femur with a 8 mm resolution, an 80 µA tube current and an 80 kV tube voltage. A Micro‐CT system (GE Healthcare Bio‐Sciences, MicroView 1.1 software) was utilized to carry out reconstruction and 3D quantitative analyses. In femora, the regions of scanning were limited to distal metaphysis and were extended about 2.0 mm from proximal tip of the primary spongiosa. For micro‐CT section, each trabecular bone region was outlined. For above regions, the endocortical bone surfaces were the boundaries to separate trabecular bone from cortical bone. The 3D indices listed below were analysed: bone mineral density (BMD), trabecular separation (Tb.Sp), proportion of bone volume in total volume (BV/TV), structure model index (SMI), trabecular thickness (Tb.Th) and the trabecular number (Tb.N).

### 2.4 RNA isolation and real‐time PCR

A miRNeasy Midi kit (Qiagen) was utilized to isolated total RNA from cultured cells and tissue samples. A High‐Capacity cDNA Archive kit (Applied Biosystems) was utilized to perform MultiScribe reverse transcription to synthesize cDNA from 1μg of total RNA. A LightCycler480 PCR system (Roche Diagnostics) was utilized along with a DNA binding SYBR Green dye (Applied Biosystems) to perform real‐time quantitative PCR to detect the relative expression of CB2, Notch1, Notch2, ALP and collagen type I mRNA. The PCR reactions were carried out at 95°C for 15 minutes for initial denaturation, followed by 50 cycles of 95°Cfor 15 seconds for denaturation, 55°C for 15 seconds for annealing and 72°C for 15 seconds for extension. The β‐actin and small RNA U6 were used as internal controls. 1.5% agarose gels were utilized to perform electrophoresis to visualize PCR products, and the comparative 2 −ΔΔCt method was utilized to calculate the relative expression of CB2 (Forward: 5'‐AGTGTTGGCTGTGCTCCTCATC‐3'; Reverse: 5'‐GTTGATGAGGCACAGCATGGAG‐3'), Notch1 (Forward: 5'‐ GGTGAACTGCTCTGAGGAGATC‐3'; Reverse: 5'‐ GGATTGCAGTCGTCCACGTTGA‐3'), Notch2 (Forward: 5'‐ GTGCCTATGTCCATCTGGATGG‐3'; Reverse: 5'‐ AGACACCTGAGTGCTGGCACAA‐3'), ALP (Forward: 5'‐ GCTGTAAGGACATCGCCTACCA‐3'; Reverse: 5'‐ GCTGTAAGGACATCGCCTACCA‐3'), and collagen type I mRNA (Forward: 5'‐ GATTCCCTGGACCTAAAGGTGC‐3'; Reverse: 5'‐ AGCCTCTCCATCTTTGCCAGCA‐3'), which were normalized to the expression of β‐actin and small RNA U6. Each reaction was repeated three times.

### 2.5 Cell culture and transfection

The RPMI culture medium (Gibco, Invitrogen) supplemented with 10% FBS (foetal bovine serum), 1% streptomycin‐penicillin (Invitrogen) and 2 mmol/L glutamine (Euroclone) was used to incubate the hBMSCs under a humidified atmosphere of 5% CO2/95% air at 37°C. On the day of transfection, when the cells reached 80% confluence, Lipofectamine 2000 (Invitrogen) was used to cotransfect the cells with pcDNA3‐NICD, CB2 shRNA and Notch1 shRNA. Each reaction was carried out in triplicate.

### 2.6 Vector construction

The coding sequence of NICD (Notch 1 intracellular coding domain) was amplified through PCR, and the PCR products were subsequently purified, double digested and cloned into a pcDNA3 vector. Direct Sanger sequencing was performed to confirm the sequence of the clone. All experiments were repeated three times.

### 2.7 Cell proliferation assay

PcDNA3‐NICD, CB2 shRNA or Notch1 shRNA was applied to hBMSCs. The 3‐(4, 5‐dimethylthiazol‐2‐yl)‐2, 5‐diphenyltetrazolium bromide (MTT) assay was utilized to analyse the proliferation of hBMSCs after 48 hours of transfection. Briefly, hBMSCs were seeded into 96‐well plates at a concentration of 3 × 10^3^ cells/well. Then, 50 μL of MTT was used to incubate the cells for 4 hours. HCL‐isopropanol was used to dissolve the formazan product, and a SpectraMax® microplate spectrophotometer (Molecular Devices, LLC) was utilized to examined the proliferation of hBMSCs based on the absorbance at 540 nm. Three independent experiments were carried out.

### 2.8 Western blot analysis

PcDNA3‐NICD, CB2 shRNA or Notch1 shRNA was applied to hBMSCs. At 48 hours post‐transfection, hBMSCs were harvested and lysed in an ice‐cold lysis buffer supplemented with 1% Triton X‐100, 130 mmol/L NaCl, 20 mmol/L Tris‐HCl [pH 8], 1 mmol/L sodium orthovanadate, 10% glycerol, 1 mmol/L PMSF, 2 mmol/L EDTA and protease inhibitors. Then, the lysates were subjected to centrifugation at 15 000× *g* for 10 minutes at 4°C, and 8%‐12% SDS‐PAGE was utilized to separate above lysates to obtain target proteins of CB2, Notch1, Notch2, ALP and collagen type I. Then, the proteins were electrophoretically transferred to polyvinylidene difluoride membranes (Millipore, Billerica, MA) for 2 hours at 90V, and TBS (150 mmol/L NaCl, 20 mmol/L Tris [pH 7.5]) containing 1% BSA was utilized to block the membranes at 25°C for 10 minutes to avoid non‐specific binding. The primary antibodies against CB2, Notch1, Notch2, ALP, collagen type I (1:8000) and anti‐β‐actin (1:10 000) were utilized to incubate the membranes at 4℃ overnight; then, the horseradish peroxidase (HRP)‐conjugated secondary antibody (1:13 000 dilution, Boster) was used to incubate the membranes for 60 minutes. The Chemi‐Doc™ XRS System (BIO‐RAD) and an immobilon Western chemiluminescent HRP Substrate (Millipore) were used to visualize the immune complexes and analyse the relative protein expression of CB2, Notch1, Notch2, ALP and collagen type I. All tests were repeated three times.

### 2.9 Immunohistochemistry

Four percentage paraformaldehyde was utilized to fix the tissue, which was then dehydrated, paraffin embedded and cut into 4μm sections. The monoclonal anti‐CB2, anti‐Notch1, anti‐Notch2, anti‐ALP, anti‐collagen type I (1:800) and anti‐β‐actin (1:1000) antibodies were utilized to incubate the sections, and then the horseradish peroxidase (HRP)‐conjugated secondary antibody (1:1500 dilution, Boster) was used to incubate the sections. A DAB (3, 3‐diaminobenzidine) substrate kit was utilized to carry out staining in accordance with manufacturer's instructions. Each experiment was performed in triplicate.

### 2.10 Statistical analysis

SPSS17.0 software package (SPSS, Inc) was utilized to conduct all statistical analyses. All data were presented as the mean ± SD (standard deviation). ANOVA (one‑way analysis of variance) was performed to compare between two groups. Three‑way ANOVA was performed to compare the differences among three groups. The *P* value of less than .05 was considered significant.

## 3 RESULTS

### 3.1 2‐AG administration up‐regulated BMD in OVX mice

**Figure 1:**
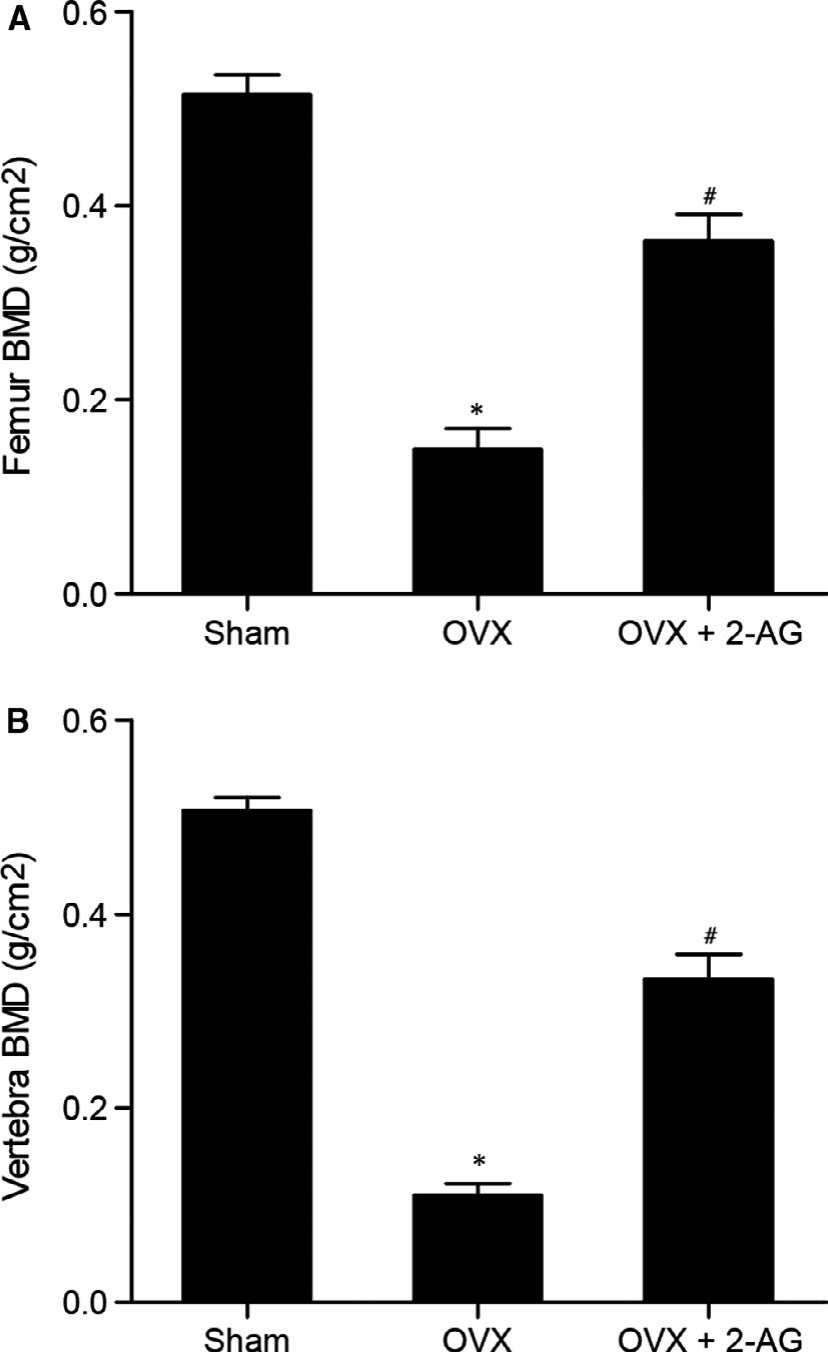
2‐AG treatment improved BMD in OVX mice, and increased BMD in femur (A) and vertebra (B) in OVX mice (*P value < .05 vs sham group; #*P* value <.05 vs OVX group; OVX: ovariectomy)

As shown in Figure 1, evident decreases in the BMD in femur (Figure 1A) and vertebra (Figure 1B) were observed in OVX mice compared with sham‐operated mice, while treatment of 2‐AG could partly restore the inhibited BMD in femur (Figure 1A) and vertebra (Figure 1B) in OVX mice, indicating that 2‐AG could up‐regulate BMD in OVX mice.

### 3.2 Effect of 2‐AG on structural properties of bone

**Figure 2:**
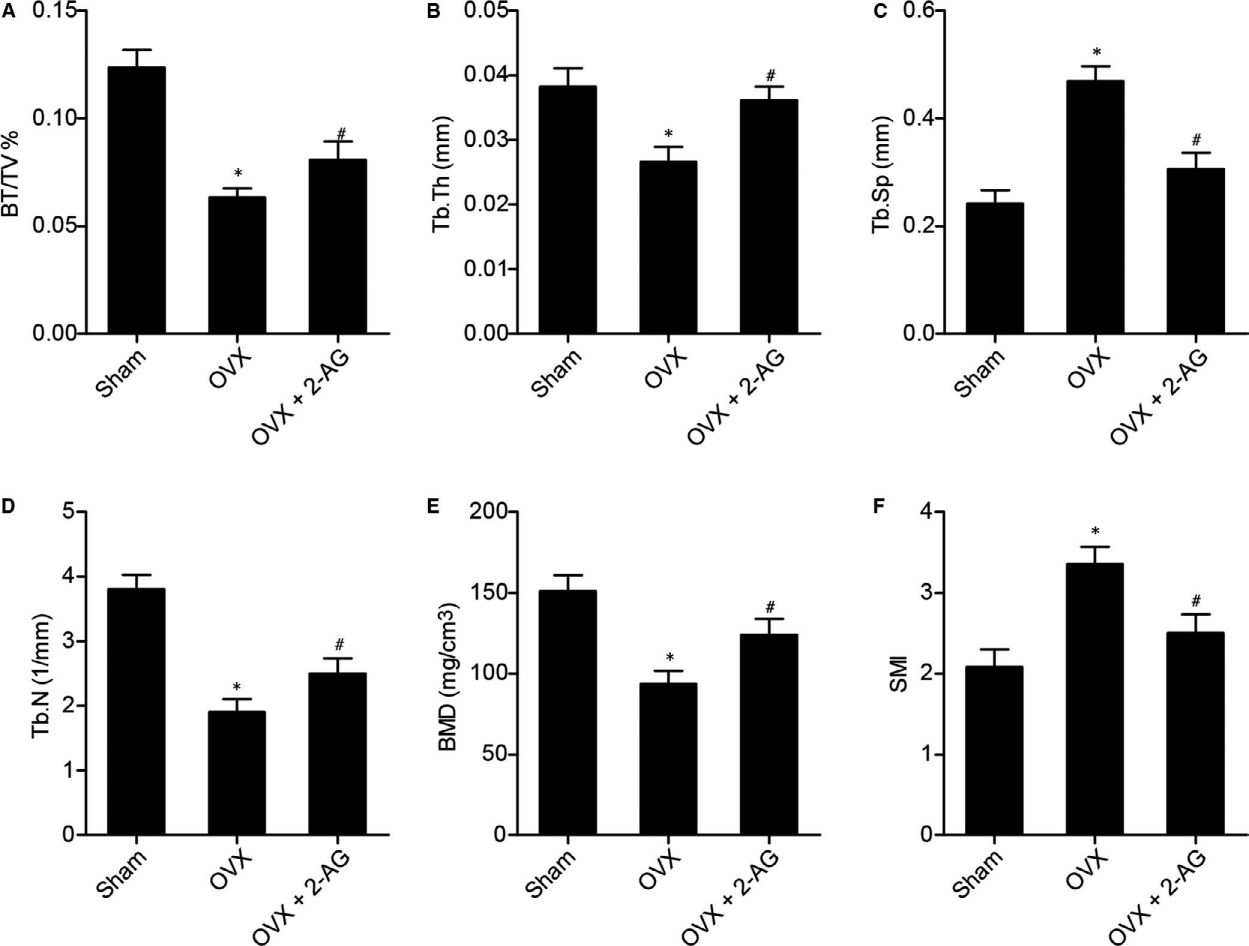
Femur bone architecture was assessed by micro‐CT (*P value <.05 vs sham group; #*P* value <.05 vs OVX group; BV/TV, proportion of bone volume in total volume; BMD, bone mineral density; SMI, structure model index; Tb.Th, trabecular thickness; Tb.Sp, trabecular separation; Tb.N, trabecular number). (A) 2‐AG increased BV/TV in OVX mice. (B) 2‐AG increased Tb.Th in OVX mice. (C) 2‐AG decreased Tb.Sp in OVX mice. (D) 2‐AG up‐regulated Tb.N in OVX mice. (E) 2‐AG treatment elevated BMD in OVX mice. (F) 2‐AG down‐regulated SMI in OVX mice

Alterations of femur bone architecture have been generally considered to be important in evaluating osteoporosis and to be associated with bone strength. To explore the effect of 2‐AG on femur bone architecture, bone microarchitecture in the distal femur was scanned using micro‐CT. As shown in Figure 2, BV/TV (Figure 2A), Tb.Th (Figure 2B), Tb.N (Figure 2D) and BMD (Figure 2E) were evidently decreased while Tb.Sp (Figure 2C) and SMI (Figure 2F) were significantly increased in the OVX group. Meanwhile, higher levels of BV/TV (Figure 2A), Tb.Th (Figure 2B), Tb.N (Figure 2D) and BMD (Figure 2E) were noted in the OVX + 2‐AG group, and lower levels of Tb.Sp (Figure 2C) and SMI (Figure 2F) were noted in the OVX + 2‐AG group.

### 3.3 Effect of 2‐AG on the Notch1 signalling pathway

**Figure 3:**
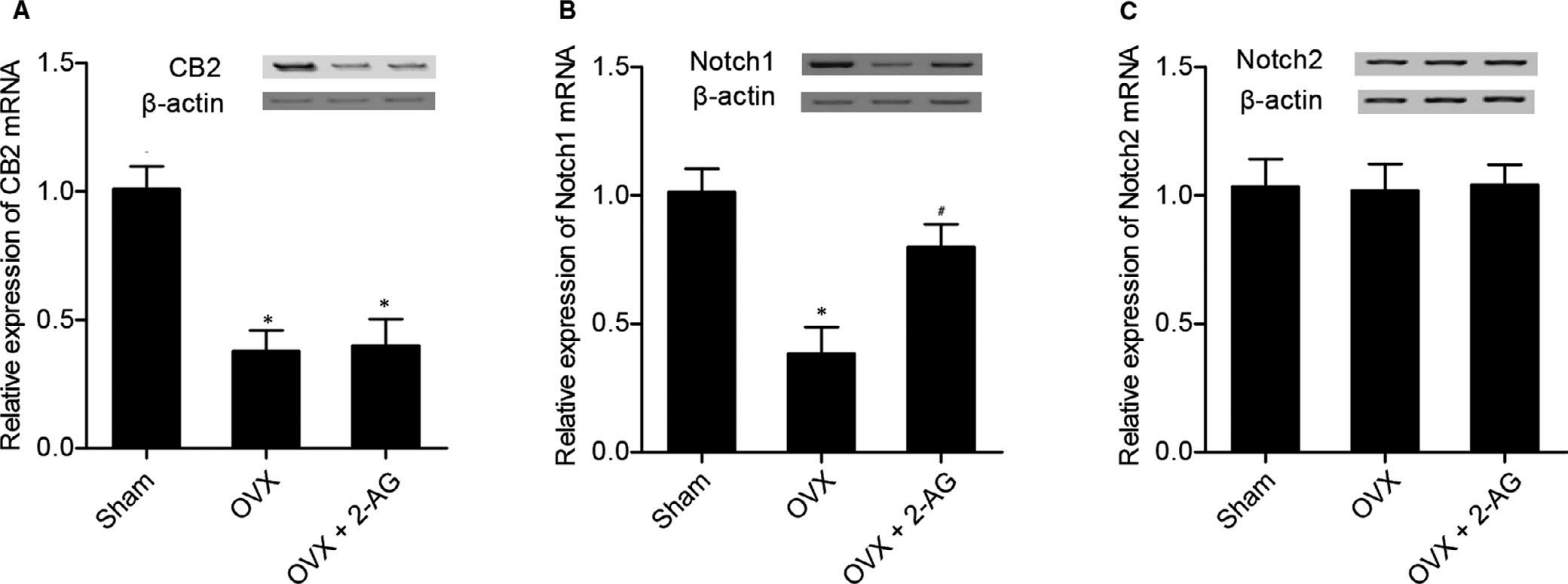
Real‐time PCR and Western blot analysis were carried out to detect the levels of CB2, Notch1 and Notch 2 among sham, OVX and OVX + 2‐AG groups (*P value <.05 vs sham group; #*P* value <.05 vs OVX group). (A) CB2 levels in OVX and OVX + 2‐AG groups were much lower than that in the sham group. (B) Notch 1 levels in OVX and OVX + 2‐AG groups were much lower than that in the sham group, and Notch 1 level in the OVX group was lower than that in the OVX + 2‐AG group. (C) Notch 2 level was comparable among sham, OVX and OVX + 2‐AG groups

Furthermore, real‐time PCR and Western blot analysis were carried out to detect the levels of CB2, Notch1 and Notch 2 among the sham, OVX and OVX + 2‐AG groups. As shown in Figure 3, CB2 (Figure 3A) levels in both OVX and OVX + 2‐AG groups were much lower than that in the sham group, while the CB2 level in the OVX group showed no obvious difference from that in the OVX + 2‐AG group. Notch 1 (Figure 3B) mRNA and protein levels in OVX and OVX + 2‐AG groups were much lower than those in the sham group, while Notch 1 level in the OVX + 2‐AG group was partly restored. Notch 2 (Figure 3C) mRNA and protein levels were comparable among sham, OVX and OVX + 2‐AG groups.

### 3.4 Effect of 2‐AG on the proliferation and differentiation of hBMSCs

**Figure 4:**
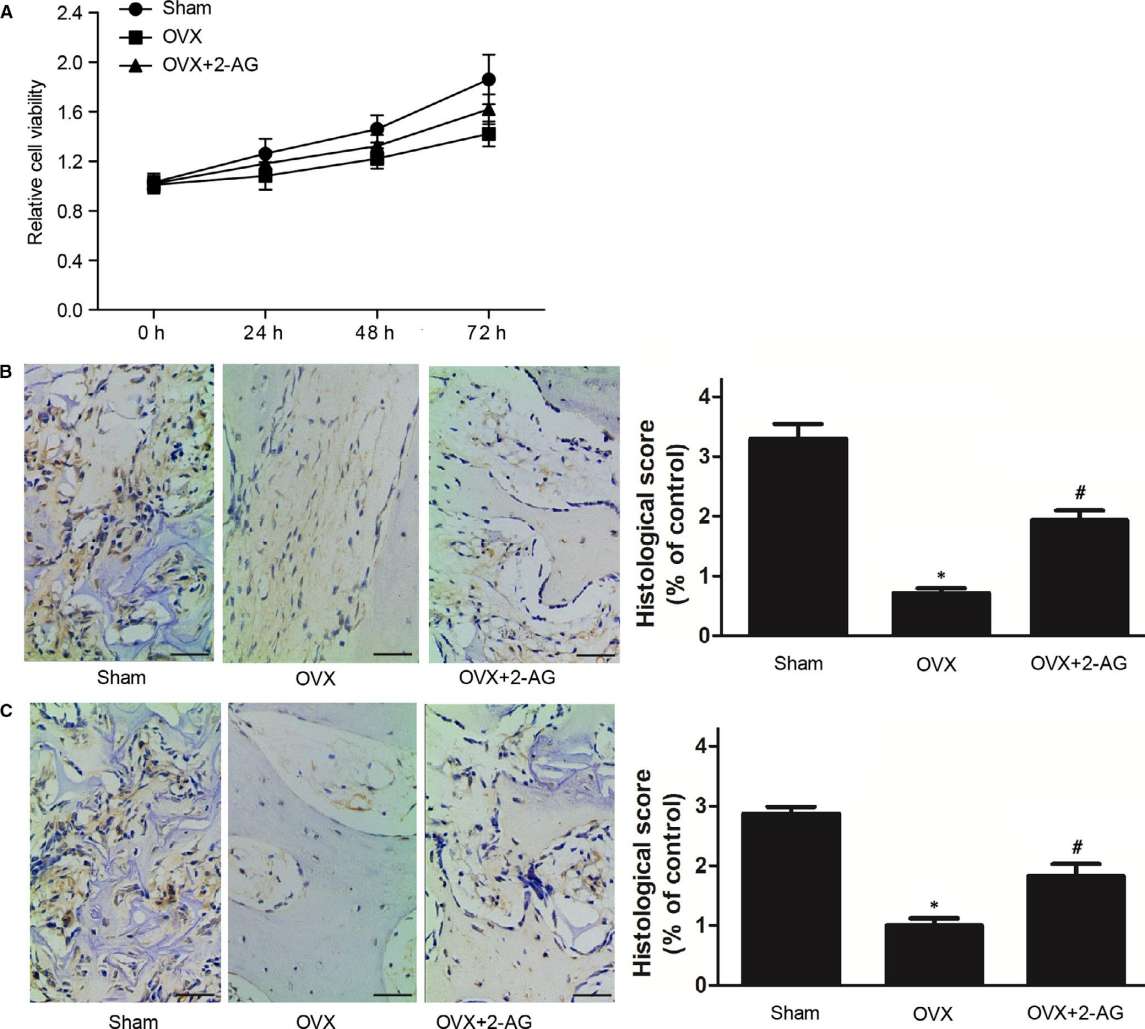
Effect of 2‐AG on the proliferation and differentiation of hBMSCs was determined using MTT assay. (A) Proliferation in sham and OVX + 2‐AG groups was much higher than that in the OVX group. (B) Collagen type I level in OVX and OVX + 2‐AG groups was much lower than that in the sham group, and collagen type I level in the OVX group was even lower. (C) ALP level in OVX and OVX + 2‐AG groups was much lower than that in the sham group, and ALP level in OVX group was even lower

MTT assay was performed and differentiation biomarkers such as ALP and collagen type I were measured to identify the effect of 2‐AG on the proliferation and differentiation of BMSCs. As shown in Figure 4A, the viability of hBMSCs in OVX and OVX + 2‐AG groups were substantially reduced. Furthermore, the viability of hBMSCs in OVX exhibited no significant difference with that in the OVX + 2‐AG group. As biomarkers of differentiation of hBMSCs, collagen type I (Figure 4B) and ALP (Figure 4C) in OVX and OVX + 2‐AG groups were much lower than those in the sham group, while the 2‐AG treatment partly restored the suppressed levels of collagen type I (Figure 4B) and ALP (Figure 4C) in the OVX group.

### 3.5 Effect of Notch1 shRNA on the expression of CB2, Notch1 and Notch2 in 2‐AG‐treated hBMSCs

**Figure 5:**
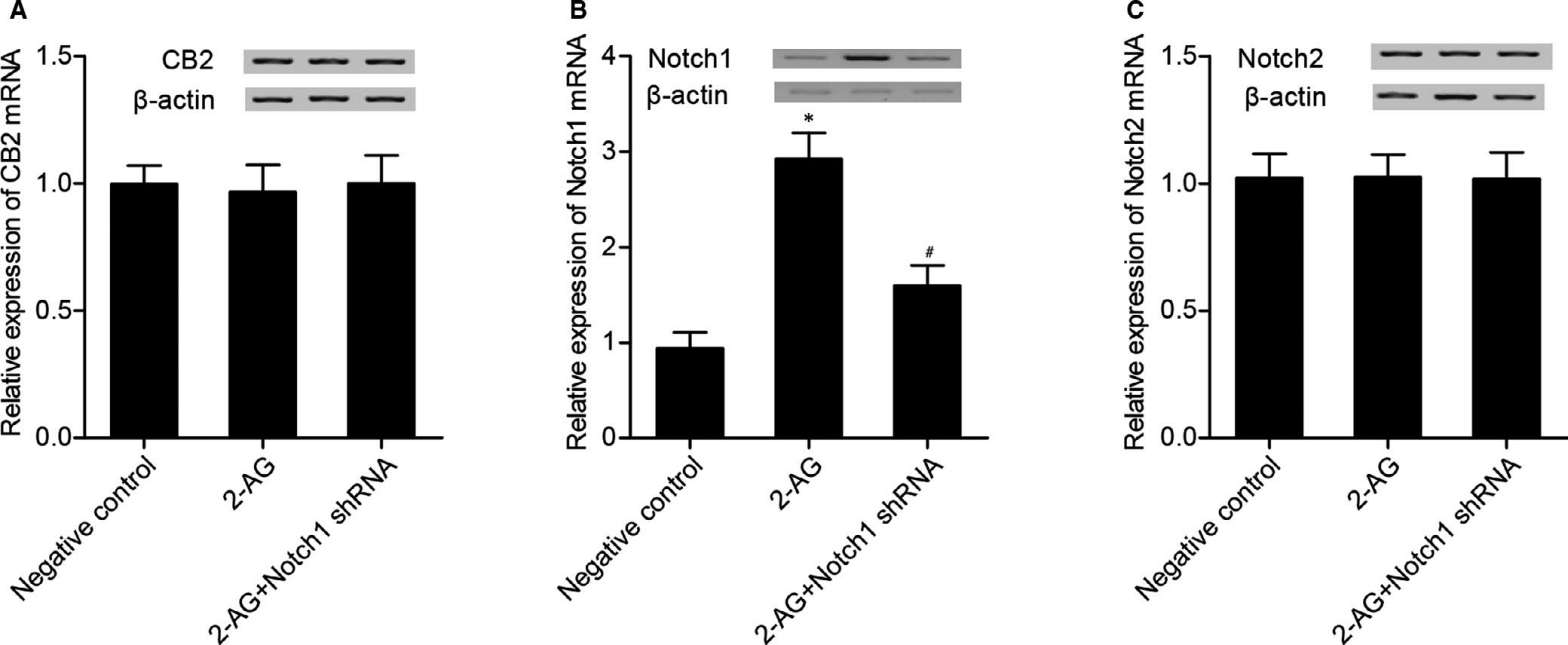
(A) Notch1 shRNA and 2‐AG had no effect on CB2 expression. (B) Notch 1 level in 2‐AG and 2‐AG + Notch1 shRNA groups was much higher than that in the NC group, and Notch 1 level in 2‐AG group was even higher than that in the 2‐AG + Notch1 shRNA group (*P value <.05 vs negative control group; #*P* value <.05 vs. 2‐AG group). (C) Notch1 shRNA and 2‐AG had no effect on Notch 2 expression

2‐AG‐treated hBMSCs were transfected with Notch1 shRNA before the levels of CB2, Notch 1 and Notch 2 were detected. As shown in Figure 5, Notch1 shRNA and 2‐AG had no effect on CB2 (Figure 5A) and Notch 2 (Figure 5C) levels. However, 2‐AG substantially increased Notch 1 expression (Figure 5B), while the transfection of Notch shRNA into 2‐AG‐treated hBMSCs obviously decreased Notch 1 expression.

### 3.6 Effects of 2‐AG and Notch 1 on the proliferation and differentiation of hBMSCs

**Figure 6:**
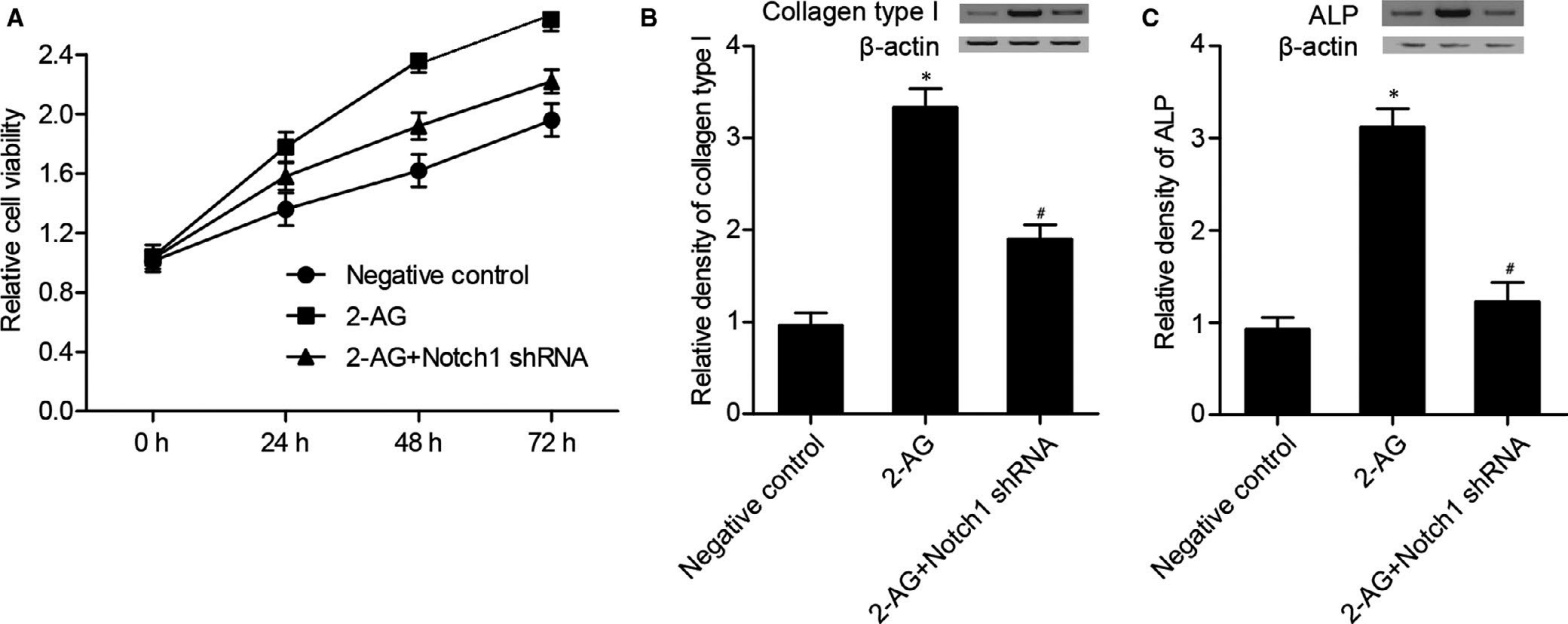
(A) Viability of hBMSCs in 2‐AG and 2‐AG + Notch 1 shRNA groups was enhanced, and the effect of 2‐AG on cell viability was stronger than 2‐AG + Notch 1 shRNA. (B) Collagen type I differentiation biomarkers such as ALP and collagen type I were measured to identify the effect of 2‐AG on the proliferation and differentiation of BMSCs. Collagen type I level in 2‐AG and 2‐AG + Notch 1 shRNA groups was much higher than that in the NC group (*P value <.05 vs negative control group; #*P* value <.05 vs 2‐AG group). (C) ALP level in 2‐AG and 2‐AG + Notch 1 shRNA groups was much higher than that in the NC group (*P value <.05 vs negative control group; #*P* value <.05 vs 2‐AG group)

As shown in Figures 6A, 2‐AG and 2‐AG + Notch 1 shRNA significantly promoted the proliferation of hBMSCs, while the effect of 2‐AG was stronger than 2‐AG + Notch 1 shRNA. Collagen type I (Figure 6B) and ALP (Figure 6C) levels in 2‐AG and 2‐AG + Notch 1 shRNA groups were much higher than those in the NC group, and collagen type I (Figure 6B) and ALP (Figure 6C) levels in 2‐AG group were even higher than those in the 2‐AG + Notch 1 shRNA group.

### 3.7 Effect of CB2 shRNA and NICD on the expression of CB2 and Notch signals.

**Figure 7:**
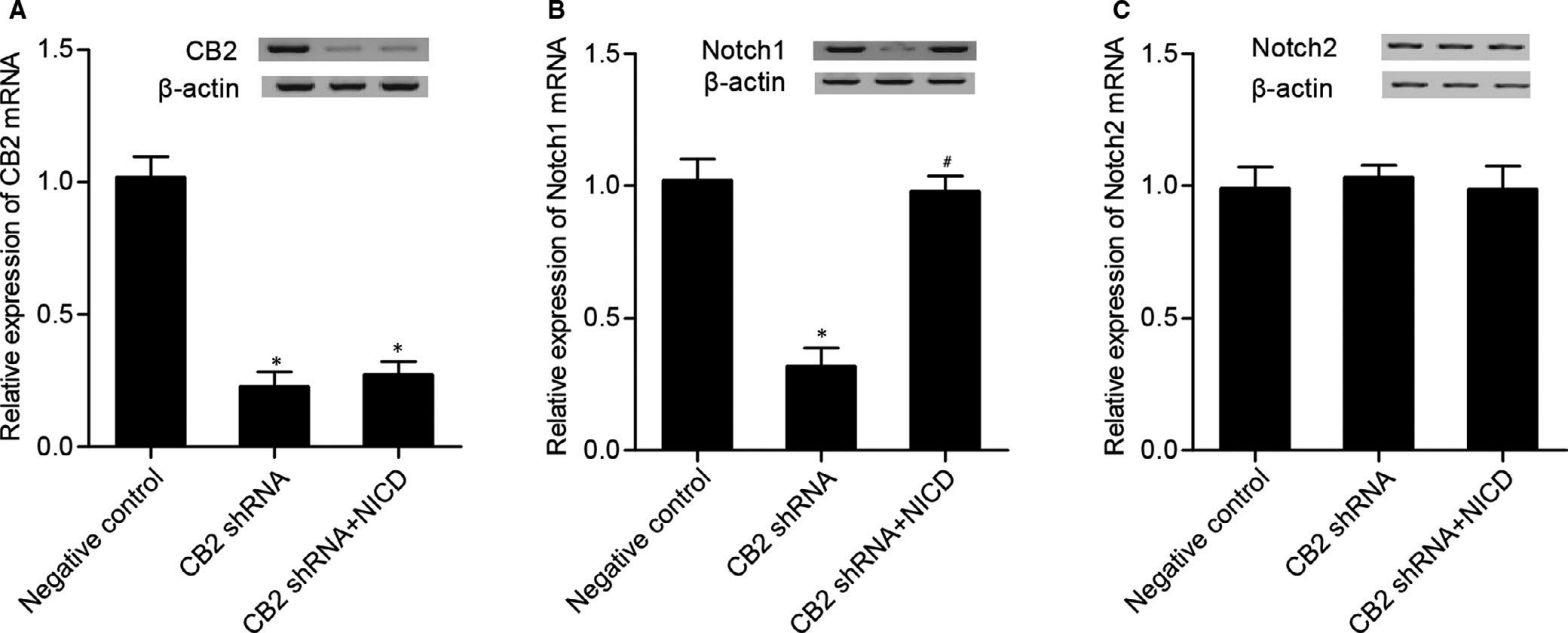
(A) CB2 levels in CB2 shRNA and CB2 shRNA + NICD groups were much lower than that in the NC group (*P value <.05 vs negative control group). (B) Notch I level in CB2 shRNA + NICD and NC groups was much higher than that in the CB2 shRNA group (*P value <.05 vs negative control group; #*P* value <.05 vs CB2 shRNA group). (C) CB2 shRNA and CB2 shRNA + NICD had no effect on Notch 2 expression

Real‐time PCR and Western blot analysis were performed to examine the levels of CB2, Notch 1 and Notch 1 in cells transfected with CB2 shRNA and CB2 shRNA + NICD. As shown in Figure 7A, both CB2 shRNA transfection and cotransfection of CB2 shRNA + NICD significantly decreased CB2 levels, and the inhibitory effect of CB2 shRNA was comparable with that of CB2 shRNA + NICD. The Notch 1 (Figure 7B) level in the CB2 shRNA group was much lower than that in the NC group, while the transfection of CB2 shRNA along with NICD almost fully restored Notch 1 level compared with the CB2 shRNA group. Finally, CB2 and NICD had no effect on Notch 2 expression.

### 3.8 Effects of CB2 shRNA and NICD on the proliferation and differentiation of hBMSCs

**Figure 8:**
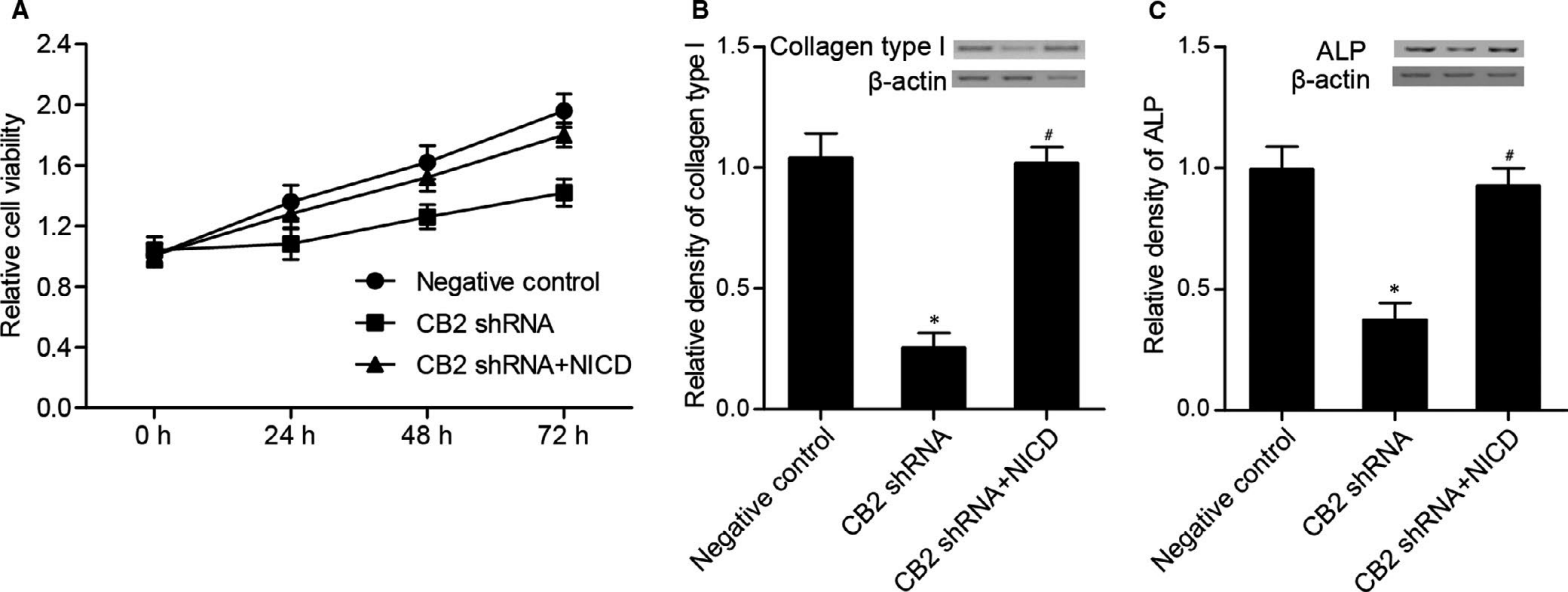
(A) Survival of hBMSCs in CB2 shRNA + NICD and NC groups was much higher than that in the CB2 shRNA group. (B) Collagen type I level in CB2 shRNA + NICD and NC groups was much higher than that in the CB2 shRNA group (*P value <.05 vs negative control group; #*P* value <.05 vs CB2 shRNA group). (C) ALP level in CB2 shRNA + NICD and NC groups was much higher than that in the CB2 shRNA group (*P value <.05 vs negative control group; #*P* value <.05 vs CB2 shRNA group)

As shown in Figure 8A, CB2 shRNA significantly suppressed the proliferation of hBMSCs, and transfection of CB2 shRNA along with NICD fully restored hBMSCs viability. CB2 shRNA significantly inhibited collagen type I (Figure 8B) and ALP (Figure 8C) expression, while the presence of NICD fully restored collagen type I and ALP expression in CB2 shRNA‐transfected cells.

## 4 DISCUSSION

Two identified cannabinoid receptors in the G‐protein coupled receptor family, CB1 and CB2, are bound and activated by endocannabinoids and their synthetic analogues.^18^ CB1 and CB2 receptors are coupled to cyclic adenosine monophosphate (cAMP) and adenylyl cyclase together with a lot of other second messengers such as PI3 Kinase/Akt and phospholipase C.^19^ CB1 is primarily expressed in the brain, while CB2 is mainly expressed in the immune cells.^20^ Recent studies, however, have shown that CB1 and CB2 receptors are also found in other organs, tissues and cells such as adipocytes and bone cells.^11^ It has been shown that 2‐AG stimulates Notch 2 while AEA stimulates Notch 1. Recently, in dendritic cells with Δ^9^‐tetrahydrocannabinol elevating the production of Jagged 1 and reducing the expression of Notch ligand Delta 4, a similar stimulation of the NSP by cannabinoids has been observed.^21^ This first suggested that cannabinoid plays a role in NS.^9^ In this study, we established an animal model of osteoporosis, which was further treated with 2‐AG. Next, we performed MTT assay and examined the differentiation of biomarkers such as ALP and collagen type I to identify the effect of 2‐AG on the proliferation and differentiation of BMSCs. Accordingly, we found that 2‐AG promoted the differentiation of hBMSCs in OVX mice.

It was shown that osteoporotic BMSCs have elevated adipogenesis, featured by an impaired leptin action.^22^ Zhang et al^23^ found the promotion of adipogenic potential and the decrease of osteogenic potential in osteoporotic BMSCs. Osteoporotic BMSCs are different from normal controls in that they have a reduced growth rate and are insensitive to the mitogenic effect of IGF‐1, which is implicated in the MAPK signalling pathway.^24^ In osteoporotic postmenopausal women, the ability of BMSCs to produce and maintain type I collagen‐rich extracellular matrix is reduced, promoting the cell differentiation of adipogenesis.^25^

Canonical NS is a well‐known modulator of stem cell self‐renewal, proliferation and differentiation.^9^ Other reports have recently found that, during mouse limb‐bud and postnatal bone development, the RBPjκ‐dependent NSP could function as a suppressor of MPC/BMSC differentiation and a significant trigger of BMSC and mesenchymal progenitor cell (MPC) proliferation.^26^ These mouse studies showed that conditional ablation of the components of NSP (HES/HEY factors, RBPjκ, NOTCH1 or NOTCH2) in early BMSCs and MPCs of the limbs led to increased osteogenic and differentiation of chondrogenesis, followed by a depleted BMSC pool.^26^ Recent data also showed that one or more of the HES/HEY NOTCH‐associated factors may trigger this BMSC/MPC suppression of differentiation.^26^ Other studies have demonstrated that persistent stimulation of the NP in cultured human BMSCs through viral infection with *NICD1* or *JAG1* inhibited differentiation of chondrogenesis.^27^ Moreover, cultured human BMSCs treated with a DAPT (*N*‐[2*S*‐(3, 5‐difluorophenyl)acetyl]‐l‐alanyl‐2‐phenyl‐1,1‐dimethylethyl ester‐glycine) suppressor had lower in vitro proliferative capacity.^28^ A previous study has shown that γ‐secretase activity can be regulated by the recruitment and dependence of the γ‐secretase complex to structures of lipid raft; hence, it is plausible that agents that disrupt or stabilize lipid raft structures may also modulate the NSP.^29^, ^30^ In this study, we investigated the relationship among 2‐AG, CB2 and Notch 1, and found that 2‐AG up‐regulated Notch 1 expression, but had no effect on CB2 expression. Furthermore, we investigated the effects of 2‐AG and Notch 1 on the proliferation and differentiation of hBMSCs, and found that 2‐AG and Notch 1 shRNA promoted and inhibited cell proliferation and differentiation, respectively.

An earlier study demonstrated that mice with CNR2 knockout had a reduced bone mass, reminiscent of human osteoporosis.^31^, ^32^ Some reports have shown the relationship between BMD and CNR2 polymorphisms.^14^ Recently, it has been shown that CNR2 gene was related to BMD in postmenopausal women in Korea.^33^ Further evidence supported the relationship between CNR2 gene and BMD. Two SNPs of rs2501431 and rs4237 in CNR2 gene may be indicative of the risk of osteoporosis in Chinese postmenopausal women.^34^ In this study, we revealed that CB2 shRNA decreased Notch I level, while NICD increased Notch I level. However, NICD had no effect on CB2 expression. In addition, we also found that CB2 shRNA inhibited cell proliferation and differentiation, while NICD promoted proliferation and differentiation of hBMSCs.

## 5 CONCLUSION

In conclusion, our results provided further evidence for the association of CB2 gene with BMD and osteoporosis. Our study revealed that CB2 inhibited Notch 1 expression, and Notch 1 served as a regulator of proliferation and differentiation of hBMSCs. Dysregulation of proliferation and differentiation of hBMSCs is believed to play an important role in the development of osteoporosis. Therefore, our study provided a deeper insight into the pathophysiology of osteoporosis and identified CB2 as a promising target for the treatment of osteoporosis.

## CONFLICT OF INTERESTS

None.

## AUTHOR CONTRIBUTIONS

**Feng Tian:** Conceptualization (equal); Investigation (equal); Project administration (equal); Validation (equal); Writing‐original draft (equal). **Hong‐tao Yang:** Formal analysis (equal); Investigation (equal); Methodology (equal); Software (equal); Writing‐original draft (equal). **Tao Huang:** Investigation (equal); Validation (equal); Visualization (equal). **Feng‐feng Chen:** Investigation (equal); Methodology (equal); Software (equal); Validation (equal). **Fu‐jun Xiong:** Conceptualization (equal); Methodology (equal); Project administration (equal); Resources (equal); Supervision (equal); Writing‐original draft (equal).

## Data Availability

The data that support the findings of this study are available from the corresponding author upon reasonable request.

## References

[1] Siris ES , Adler R , Bilezikian J , et al. The clinical diagnosis of osteoporosis: a position statement from the National Bone Health Alliance Working Group. Osteoporos Int. 2014;25(5):1439‐1443. 24577348 10.1007/s00198-014-2655-z PMC3988515

[2] Chen TL . Inhibition of growth and differentiation of osteoprogenitors in mouse bone marrow stromal cell cultures by increased donor age and glucocorticoid treatment. Bone. 2004;35(1):83‐95. 15207744 10.1016/j.bone.2004.03.019

[3] Charoenpanich A , Wall ME , Tucker CJ , et al. Cyclic tensile strain enhances osteogenesis and angiogenesis in mesenchymal stem cells from osteoporotic donors. Tissue Eng Part A. 2014;20:67‐78. 23927731 10.1089/ten.tea.2013.0006 PMC3875187

[4] Bray SJ . Notch signalling: a simple pathway becomes complex. Nat Rev Mol Cell Biol. 2006;7:678‐689. 16921404 10.1038/nrm2009

[5] Ugarte F , Ryser M , Thieme S , et al. Notch signaling enhances osteogenic differentiation while inhibiting adipogenesis in primary human bone marrow stromal cells. Exp Hematol. 2009;37(867–875):e861. 10.1016/j.exphem.2009.03.007 19540436

[6] Yanjie J , Jiping S , Yan Z , Xiaofeng Z , Boai Z , Yajun L . Effects of Notch‐1 signalling pathway on differentiation of marrow mesenchymal stem cells into neurons in vitro. NeuroReport. 2007;18:1443‐1447. 17712271 10.1097/WNR.0b013e3282ef7753

[7] Xu J , Liu X , Chen J , et al. Simvastatin enhances bone marrow stromal cell differentiation into endothelial cells via notch signaling pathway. Am J Physiol Cell Physiol. 2009;296:C535‐543. 19109527 10.1152/ajpcell.00310.2008 PMC2660258

[8] Mackie K . Mechanisms of CB1 receptor signaling: endocannabinoid modulation of synaptic strength. Int J Obes. 2006;30(Suppl 1):S19‐23. 10.1038/sj.ijo.0803273 16570100

[9] Frampton G , Coufal M , Li H , Ramirez J , DeMorrow S . Opposing actions of endocannabinoids on cholangiocarcinoma growth is via the differential activation of Notch signaling. Exp Cell Res. 2010;316:1465‐1478. 20347808 10.1016/j.yexcr.2010.03.017 PMC2872061

[10] Bertrand N , Castro DS , Guillemot F . Proneural genes and the specification of neural cell types. Nat Rev Neurosci. 2002;3:517‐530. 12094208 10.1038/nrn874

[11] Ofek O , Karsak M , Leclerc N , et al. Peripheral cannabinoid receptor, CB2, regulates bone mass. Proc Natl Acad Sci U S A. 2006;103:696‐701. 16407142 10.1073/pnas.0504187103 PMC1334629

[12] Sophocleous A , Landao‐Bassonga E , Van't Hof RJ , Idris AI , Ralston SH . The type 2 cannabinoid receptor regulates bone mass and ovariectomy‐induced bone loss by affecting osteoblast differentiation and bone formation. Endocrinology. 2011;152:2141‐2149. 21447627 10.1210/en.2010-0930

[13] Karsak M , Cohen‐Solal M , Freudenberg J , et al. Cannabinoid receptor type 2 gene is associated with human osteoporosis. Hum Mol Genet. 2005;14:3389‐3396. 16204352 10.1093/hmg/ddi370

[14] Fan JZ , Yang L , Meng GL , et al. Estrogen improves the proliferation and differentiation of hBMSCs derived from postmenopausal osteoporosis through notch signaling pathway. Mol Cell Biochem. 2014;392:85‐93. 24752351 10.1007/s11010-014-2021-7 PMC4053611

[15] Liao L , Su X , Yang X , et al. TNF‐alpha inhibits FoxO1 by upregulating miR‐705 to aggravate oxidative damage in bone marrow‐derived mesenchymal stem cells during osteoporosis. Stem Cells. 2016;34:1054‐1067. 26700816 10.1002/stem.2274

[16] Kim D , Lim S , Park M , et al. Ubiquitination‐dependent CARM1 degradation facilitates Notch1‐mediated podocyte apoptosis in diabetic nephropathy. Cell Signal. 2014;26:1774‐1782. 24726896 10.1016/j.cellsig.2014.04.008

[17] Zhang N , Gui Y , Qiu X , et al. DHEA prevents bone loss by suppressing the expansion of CD4(+) T cells and TNFa production in the OVX‐mouse model for postmenopausal osteoporosis. Biosci Trends. 2016;10(4):277‐287. 27464762 10.5582/bst.2016.01081

[18] Pertwee RG , Ross RA . Cannabinoid receptors and their ligands. Prostaglandins Leukot Essent Fatty Acids. 2002;66:101‐121. 12052030 10.1054/plef.2001.0341

[19] Sanchez MG , Ruiz‐Llorente L , Sanchez AM , Diaz‐Laviada I . Activation of phosphoinositide 3‐kinase/PKB pathway by CB(1) and CB(2) cannabinoid receptors expressed in prostate PC‐3 cells. Involvement in Raf‐1 stimulation and NGF induction. Cell Signal. 2003;15:851‐859. 12834810 10.1016/s0898-6568(03)00036-6

[20] Reddy V , Grogan D , Ahluwalia M , et al. Targeting the endocannabinoid system: a predictive, preventive, and personalized medicine‐directed approach to the management of brain pathologies. EPMA J. 2020;11(2):217‐250. 32549916 10.1007/s13167-020-00203-4 PMC7272537

[21] Newton CA , Chou PJ , Perkins I , Klein TW . CB(1) and CB(2) cannabinoid receptors mediate different aspects of delta‐9‐tetrahydrocannabinol (THC)‐induced T helper cell shift following immune activation by Legionella pneumophila infection. J Neuroimmune Pharmacol. 2009;4:92‐102. 18792785 10.1007/s11481-008-9126-2

[22] Astudillo P , Rios S , Pastenes L , Pino AM , Rodriguez JP . Increased adipogenesis of osteoporotic human‐mesenchymal stem cells (MSCs) characterizes by impaired leptin action. J Cell Biochem. 2008;103:1054‐1065. 17973271 10.1002/jcb.21516

[23] Zhang Y , Ma C , Liu X , et al. Epigenetic landscape in PPARgamma2 in the enhancement of adipogenesis of mouse osteoporotic bone marrow stromal cell. Biochim Biophys Acta. 2015;1852:2504‐2516. 26319419 10.1016/j.bbadis.2015.08.020

[24] Fallahnezhad S , Piryaei A , Darbandi H , et al. Effect of low‐level laser therapy and oxytocin on osteoporotic bone marrow‐derived mesenchymal stem cells. J Cell Biochem. 2018;119(1):983‐997. 28681933 10.1002/jcb.26265

[25] Rodriguez JP , Montecinos L , Rios S , Reyes P , Martinez J . Mesenchymal stem cells from osteoporotic patients produce a type I collagen‐deficient extracellular matrix favoring adipogenic differentiation. J Cell Biochem. 2000;79:557‐565. 10996846 10.1002/1097-4644(20001215)79:4<557::aid-jcb40>3.0.co;2-h

[26] Dong Y , Jesse AM , Kohn A , et al. RBPjkappa‐dependent Notch signaling regulates mesenchymal progenitor cell proliferation and differentiation during skeletal development. Development. 2010;137:1461‐1471. 20335360 10.1242/dev.042911 PMC2853848

[27] Oldershaw RA , Tew SR , Russell AM , et al. Notch signaling through Jagged‐1 is necessary to initiate chondrogenesis in human bone marrow stromal cells but must be switched off to complete chondrogenesis. Stem Cells. 2008;26:666‐674. 18192230 10.1634/stemcells.2007-0806

[28] Vujovic S , Henderson SR , Flanagan AM , Clements MO . Inhibition of gamma‐secretases alters both proliferation and differentiation of mesenchymal stem cells. Cell Prolif. 2007;40:185‐195. 17472726 10.1111/j.1365-2184.2007.00426.x PMC6495689

[29] DeMorrow S , Glaser S , Francis H , et al. Opposing actions of endocannabinoids on cholangiocarcinoma growth: recruitment of Fas and Fas ligand to lipid rafts. J Biol Chem. 2007;282:13098‐13113. 17329257 10.1074/jbc.M608238200

[30] Gamerdinger M , Clement AB , Behl C . Effects of sulindac sulfide on the membrane architecture and the activity of gamma‐secretase. Neuropharmacology. 2008;54:998‐1005. 18359496 10.1016/j.neuropharm.2008.02.009

[31] Rossi F , Bellini G , Luongo L , et al. The endovanilloid/endocannabinoid system: a new potential target for osteoporosis therapy. Bone. 2011;48:997‐1007. 21237298 10.1016/j.bone.2011.01.001

[32] Devoto M , Shimoya K , Caminis J , et al. First‐stage autosomal genome screen in extended pedigrees suggests genes predisposing to low bone mineral density on chromosomes 1p, 2p and 4q. Eur J Hum Genet. 1998;6:151‐157. 9781060 10.1038/sj.ejhg.5200169

[33] Woo JH , Kim H , Kim JH , Kim JG . Cannabinoid receptor gene polymorphisms and bone mineral density in Korean postmenopausal women. Menopause. 2015;22:512‐519. 25268406 10.1097/GME.0000000000000339

[34] Zhang C , Ma J , Chen G , Fu D , Li L , Li M . Evaluation of common variants in CNR2 gene for bone mineral density and osteoporosis susceptibility in postmenopausal women of Han Chinese. Osteoporos Int. 2015;26:2803‐2810. 26055357 10.1007/s00198-015-3195-x

